# mlr3proba: an R package for machine learning in survival analysis

**DOI:** 10.1093/bioinformatics/btab039

**Published:** 2021-02-01

**Authors:** Raphael Sonabend, Franz J. Király, Andreas Bender, Bernd Bischl, Michel Lang

**Affiliations:** Department of Statistical Science, University College London, London WC1E 6BT, UK; Department of Statistical Science, University College London, London WC1E 6BT, UK; Department of Statistics, LMU Munich, Munich 80539, Germany; Department of Statistics, LMU Munich, Munich 80539, Germany; Department of Statistics, LMU Munich, Munich 80539, Germany

## Abstract

**Summary:**

As machine learning has become increasingly popular over the last few decades, so too has the number of machine-learning interfaces for implementing these models. Whilst many R libraries exist for machine learning, very few offer extended support for survival analysis. This is problematic considering its importance in fields like medicine, bioinformatics, economics, engineering and more. **mlr3proba** provides a comprehensive machine-learning interface for survival analysis and connects with **mlr3**’s general model tuning and benchmarking facilities to provide a systematic infrastructure for survival modelling and evaluation.

**Availability and implementation:**

mlr3proba is available under an LGPL-3 licence on CRAN and at https://github.com/mlr-org/mlr3proba, with further documentation at https://mlr3book.mlr-org.com/survival.html.

## 1 Introduction

Survival analysis is the field of statistics concerned with the estimation of time-to-event distributions while accounting for censoring and truncation. **mlr3proba** introduces survival modelling to the **mlr3** (Lang *et al.*, 2019a) ecosystem of machine-learning packages. By utilizing a probabilistic supervised learning (Gressmann *et al.*, 2018) framework **mlr3proba** allows for multiple survival analysis predictions: predicting the time to an event, the probability of an event over time and the relative risk of an event. **mlr3proba** includes an extensive collection of classical and machine-learning models and many specialized survival measures.

The R programming language (R Core Team, 2020) provides extensive support for both survival analysis and machine learning via its core functionality and through open-source add-on packages available from CRAN and Bioconductor. **mlr3proba** leverages these packages by connecting a multitude of machine-learning models and measures for survival analysis. **mlr3proba** currently supports simulation of survival data, classical survival models, prediction of survival distributions by machine learning and support for high-dimensional data. Interfacing other packages in the **mlr3** family provides functionality for optimization, tuning, benchmarking and more.

## 2 Implemented functionality

A standard pipeline for survival analysis consists of: (i) defining a survival task as a set of features and survival outcome (time until the event and a censoring indicator); (ii) training a model on survival data, with the possibility of optimization via tuning of hyper-parameters; (iii) making predictions from the trained model on new data; and (iv) evaluating the quality of predictions with survival-specific measures, possibly including visualization.

**mlr3proba** streamlines this process by: (i) standardizing survival tasks, with the Surv object from the **survival** (Therneau, 2015) package, into a single object capable of handling left-, interval- and right-censoring (TaskSurv); (ii) unifying all survival learners (LearnerSurv*) with (iii) prediction objects that clearly distinguish model prediction types (PredictionSurv); and (iv) unifying survival measures for different survival prediction types (MeasureSurv*).

Careful design and documentation of models and measures clearly demonstrate the predictions that can be made by models or evaluated by measures. Each model can predict one or more of: response—a survival time, distr—a survival distribution, crank—a relative risk ranking, and lp—a linear predictor. distr predictions are cast into standardized distribution objects using the **distr6** package (Sonabend and Kiraly, 2019), which allows clean post-processing, such as predicting survival and hazard functions, amongst other uses.

Any survival model implemented in **mlr3proba** can be tuned via **mlr3tuning** (Lang *et al.*, 2019b), which includes several tuning methods (grid search, random search, generalized annealing and more) and termination criteria (based on iterations, runtime and more) for nested resampling and optimization on any survival measure. Additionally, all survival tasks and models can make use of **mlr3pipelines** (Binder *et al.*, 2020) for pre-processing, such as feature selection and variable encoding, and post-processing, such as prediction compositions (see below). Full details for these methods are available in the mlr3book (https://mlr3book.mlr-org.com).

## 3 Implemented classes

### 3.1 Learners

More than 20 survival learners are currently implemented. These range from classical statistical models to machine learning methods. For the former, the ‘usual’ semi- and fully-parametric models are implemented, such as Cox PH (Cox, 1972) and AFT models, as well as more advanced flexible spline methods (Royston and Parmar, 2002) and penalized regression. Machine-learning methods include random survival forests (Ishwaran *et al.*, 2008) (conditional inference, relative risk and log-rank splitting), gradient boosting machines (with multiple optimization methods) (Buhlmann and Hothorn, 2007), Van Belle’s support vector machines (Van Belle *et al.*, 2011) and artificial neural networks (Kvamme *et al.*, 2019) (including DeepSurv, DeepHit and Cox-Time). Inclusion of Python-implemented survival neural networks via **survivalmodels** (Sonabend, 2020) allows efficient cross-platform comparison of models.

### 3.2 Measures

For comparison of different models, 19 survival measures are implemented in **mlr3proba**. These include quantitative calibration measures, such as van Houwelingen’s *β* (Van Houwelingen, 2000), and visual comparisons of average distribution prediction to Kaplan–Meier. Implemented discrimination metrics include several measures of both concordance [e.g. Harrell *et al.* (1982) and Uno *et al.* (2011)] and time-dependent AUCs (Heagerty *et al.*, 2000). Scoring rules are also implemented including the log-loss, integrated log-loss, integrated Graf (or Brier) score (Graf *et al.*, 1999) and the Schmid/absolute score (Schmid *et al.*, 2011). Several of these are implemented directly in **mlr3proba** with an **Rcpp** (Eddelbuettel and Francois, 2011) implementation for fast and reliable performance.

### 3.3 Pipelines

Pipelines provide a way to combine multiple pre- and post-processing steps into an object that can be treated as a learner. Such pipelines can include general and survival-specific components. One particular use case is the (re-)casting of one prediction type to another. There are several different possible predictions that could be made by a survival learner that are not directly comparable, e.g. a relative risk cannot be directly compared to a survival distribution. Therefore **mlr3proba** extends the capabilities of any survival model by including pipelines that transform one prediction type to another. The distrcompositor pipeline transforms lp or crank predictions into distr predictions. Users have the option to specify the baseline distribution estimator (any learner implemented in **mlr3proba**) and the model form (proportional hazards, accelerated failure time or proportional odds). Another useful pipeline is the crankcompositor, this transforms a distr prediction into a crank and/or response prediction using some summary measure of the distribution, e.g. the mean or median. Obtaining a survival time prediction from a distribution is simply a case of wrapping the model in the crankcompositor pipeline. By combining these two pipelines, any model in **mlr3proba** can make any prediction type. The **mlr3pipelines** functionality allows tuning of these and further pipelines to find the optimal parameters for these compositions.

## 4 Related work

There are an increasing number of machine-learning packages across programming languages, including **caret** (Kuhn, 2008), **mlr** (Bischl *et al.*, 2016), **tidymodels** (Kuhn and Wickham, 2020) and **scikit-learn** (Pedregosa *et al.*, 2011). However, functionality for survival analysis has been mostly limited to ‘classical’ statistical models with relatively few packages supporting a machine-learning framework. R ships with the package **survival** (Therneau, 2015), which supports left-, interval-, and right-censoring, competing risks, time-dependent models, stratification and model evaluation. However, the package is limited to classical statistical models, with no support for machine learning and limited support for formal comparison or non-linear models. The Python equivalent to this package is **lifelines** (Davidson-Pilon *et al.*, 2020), which is again limited to a few classical models. **pec** (Mogensen *et al.*, 2014) implements no models itself but instead interfaces with many different survival packages to create survival probability predictions. The package’s main focus is on model evaluation via prediction error curves (‘pec’s) with little support for model building/training and predicting. **skpro** (Gressmann *et al.*, 2018) is a probabilistic supervised learning interface in Python. **skpro** extends the **scikit-learn** (Pedregosa *et al.*, 2011) interface to probabilistic models and appears to be the only package (in any language) dedicated to domain-agnostic probabilistic supervised learning. The interface provides an infrastructure for machine learning based survival analysis with design choices influencing **mlr3proba**, but **skpro** does not currently support survival models. **pysurvival** (Fotso *et al.*, 2019) is another Python package, which implements classical and machine-learning survival analysis models. The package has the advantage of being able to natively leverage specific neural network survival models, which are almost exclusively implemented in Python. Whilst not directly interfacing the **scikit–learn** interface, the package introduces unified functions for model fitting, predicting and evaluation. **scikit-survival** (Pölsterl, 2020) builds directly on **scikit-learn** to implement a few survival models and measures in a machine-learning framework. Unlike **pysurvival**, no neural networks are included, thus the two packages complement each other well.

## 5 Future developments

As of now, the package is limited to the single-event, right-censored setting. This is largely a limitation of the current implementations of the underlying learners. Future developments will focus on extensions to: stratified models, time-varying effects, left-censoring/truncation, interval censoring, competing risks and multi-state models. A recently proposed framework could be used to support most of these tasks without modification of the underlying learners (Bender *et al.*, 2020). Some extensions, however, might require updates to the learners. The near-future roadmap includes:

1. Expanding TaskSurv to accommodate the settings above.
2. Extending learners to handle (some of) the more complex settings.
3. Adding a learner-agnostic reduction pipeline for competing risks.

## 6 Example

The example below demonstrates how to benchmark three survival models and make use of the distribution compositor. Line 1: essential packages are loaded, **mlr3proba** always requires **mlr3**. Line 2: extra packages are loaded, **mlr3learners** is required for the xgboost learner and **mlr3pipelines** is required for the distribution composition. Lines 3–4: Kaplan–Meier and Cox PH learners are initialized with default parameters. Lines 5–7: the XGBoost learner, which does not provide predictions for the survival probability, is wrapped in the distrcompositor pipeline to transform its ranking prediction to a probabilistic prediction. Line 8: learners are combined into a list for use in the benchmark function. Lines 9–11: a task is created from a subset of the rats dataset from **survival**, the outcome is specified with the ‘time’ and ‘event’ arguments. Line 12: a three-fold cross-validation resampling scheme is specified. Line 13: the infrastructure for the experiment is automatically determined by supplying the task(s), learners and resampling method. Line 14: learners are resampled according to the chosen scheme and benchmarked. Line 15: predictions are aggregated over all folds and scored with the integrated log-loss to provide a final comparison.

> library(mlr3); library(mlr3proba)

> library(mlr3learners); library(mlr3pipelines)

> kaplan = lrn( ‘ surv.kaplan ’ )

> cox = lrn( ‘ surv.coxph ’ )

> xgb = ppl( ‘ distrcompositor ’ ,

+ learner = lrn( ‘ surv.xgboost ’ ),

+ estimator = ‘ kaplan ’ , form = ‘ ph ’ )

> learners = list(cox, kaplan, xgb)

> task = TaskSurv$new(id = ‘ rats ’ ,

+ backend = survival::rats[ , 1:4],

+ time = ‘ time ’ , event = ‘ status ’ )

> resample = rsmp( ‘ cv ’ , folds = 3)

> design = benchmark_grid(task, learners, resample)

> bm = benchmark(design)

> bm$aggregate(msr( ‘ surv.intlogloss ’ ))

## Funding

R.S. receives a PhD stipend from the Engineering and Physical Sciences Research Council [EP/R513143/1]. A.B. and M.L. are funded by the German Federal Ministry of Education and Research (BMBF) under grant No. 01IS18036A and by Deutsche Forschungsgemeinschaft (DFG) within the Collaborative Research Center SFB 876 ‘Providing Information by Resource-Constrained Analysis’.

*Conflict of Interest*: none declared.
